# Deficiency of the clock gene *Bmal1* affects neural progenitor cell migration

**DOI:** 10.1007/s00429-018-1775-1

**Published:** 2018-10-19

**Authors:** Amira A. H. Ali, Beryl Schwarz-Herzke, Shakila Mir, Benita Sahlender, Marion Victor, Boris Görg, Martin Schmuck, Katharina Dach, Ellen Fritsche, Andreas Kremer, Charlotte von Gall

**Affiliations:** 1 Institute of Anatomy II, Medical Faculty, Heinrich Heine University, Moorenstrasse 5, 40225 Düsseldorf, Germany; 2 Clinic for Gastroenterology, Hepatology and Infectiology, Medical Faculty, Heinrich Heine University, Moorenstrasse 5, 40225 Düsseldorf, Germany; 3 Leibniz Research Institute for Environmental Medicine, Modern Risk Assessment and Sphere Biology Group, Auf’m Hennekamp 50, 40225 Düsseldorf, Germany; 4 Department of Bioinformatics, Erasmus University Medical Center Rotterdam, 3015CN Rotterdam, The Netherlands

**Keywords:** Circadian, Clock genes, Bmal1, Rostral migratory stream, Subventricular zone, Catalase, Hydrogen peroxide, Filopodia, Cytoskeleton, p-Cofilin, RNA oxidation

## Abstract

We demonstrate the impact of a disrupted molecular clock in Bmal1-deficient (Bmal1^−/−^) mice on migration of neural progenitor cells (NPCs). Proliferation of NPCs in rostral migratory stream (RMS) was reduced in Bmal1^−/−^ mice, consistent with our earlier studies on adult neurogenesis in hippocampus. However, a significantly higher number of NPCs from Bmal1^−/−^ mice reached the olfactory bulb as compared to wild-type littermates (Bmal1^+/+^ mice), indicating a higher migration velocity in Bmal1^−/−^ mice. In isolated NPCs from Bmal1^−/−^ mice, not only migration velocity and expression pattern of genes involved in detoxification of reactive oxygen species were affected, but also RNA oxidation of catalase was increased and catalase protein levels were decreased. Bmal1^+/+^ migration phenotype could be restored by treatment with catalase, while treatment of NPCs from Bmal1^+/+^ mice with hydrogen peroxide mimicked Bmal1^−/−^ migration phenotype. Thus, we conclude that Bmal1 deficiency affects NPC migration as a consequence of dysregulated detoxification of reactive oxygen species.

**Electronic supplementary material:**

The online version of this article (10.1007/s00429-018-1775-1) contains supplementary material, which is available to authorized users.

## Introduction

Neurogenesis plays an important role in neuronal plasticity even in the adult brain. The subventricular zone (SVZ) of the lateral ventricles represents the most extensive neurogenic niches within the adult brain (Lois and Alvarez-Buylla 1994; Lim and Alvarez-Buylla 2016). It gives rise to neural progenitor cells (NPCs) which generate primarily committed neural progenitor that migrate tangentially along the rostral extension of the SVZ toward the olfactory bulb, forming the rostral migratory stream (RMS) (Lois and Alvarez-Buylla 1994; Doetsch and Alvarez-Buylla 1996; Jankovski and Sotelo 1996; Gritti et al. 2002; Lois et al. 1996). Within the RMS, NPCs form chains and continue to proliferate while migrating (Gritti et al. 2002; Wichterle et al. 1997). In mice, this considerable distance from the SVZ to the olfactory bulb of up to 5 mm is traversed by NPCs within 4–6 days (Lois and Alvarez-Buylla 1994). The migrating chains of NPCs in the RMS are surrounded by astrocytes that form glial tubes directing the migrating NPCs towards the olfactory bulb (Doetsch and Alvarez-Buylla 1996; Jankovski and Sotelo 1996; Lois et al. 1996). In the white matter of the olfactory bulb, the NPCs detach from the chains and change their direction and migrate radially to the cortex of the olfactory bulb. Finally, they differentiate into interneurons within the granule cell layer and the glomerular layer and integrate into preexisting neuronal networks (Whitman and Greer 2007; Carleton et al. 2003; Panzanelli et al. 2009). These adult-born interneurons play an important role in odor information processing and display a high degree of synaptic plasticity (Livneh et al. 2014). Efficient migration of NPCs within the RMS is a multifactorial process including intrinsic NPC properties as well as dynamic interactions between NPCs and glial tube astrocytes (Kaneko et al. 2010) and chemoattraction (Goldman and Luskin 1998).The circadian system provides an internal time keeping system to coordinate physiology and behavior within the 24 h solar day (Korf and von Gall 2012). The circadian clockwork is composed of two interlocked transcription/translation feedback loops of clock genes generating circadian rhythms in gene expression. The transcription factors brain and muscle Arnt-like protein1 (BMAL1) and circadian locomotor output cycles (CLOCK) heterodimerize, bind to E-box within gene promoters and enhance the transcription of genes encoding for clock genes such as Period (*Per*) and Cryptochrome (*Cry*) as well as of clock-controlled genes. PER and CRY proteins translocate into the nucleus, heterodimerize and inhibit CLOCK:BMAL1-mediated transcription and consequently their own expression. In addition, CLOCK:BMAL1 complex activates the transcription of nuclear receptors, REV-ERBa and RORa which regulate Bmal1 transcription (Ko and Takahashi 2006; Reppert and Weaver 2002). This molecular clockwork controls rhythmic gene expression and thus rhythmic cellular and organ function. BMAL1 is an essential component in the molecular clockwork as targeted deletion of the core clock gene Bmal1 leads to a loss of circadian rhythms in physiology and behavior. Moreover, Bmal1 is crucial for cellular redox homeostasis as Bmal1-deficient (Bmal1^−/−^) mice have increased levels of cellular oxidative stress resulting in reduced life span and premature aging (Kondratov et al. 2006). Bmal1 deficiency also affects cerebral redox homeostasis and is associated with cardinal symptoms of neurodegeneration (Musiek et al. 2013) as well as impaired learning and memory formation (Kondratova et al. 2010). Importantly, Bmal1 deficiency affects proliferation and differentiation of neuronal progenitor cells in the subgranular zone of the hippocampus (Malik et al. 2015) (Ali et al. 2015; Bouchard-Cannon et al. 2013). However, little is known about the effects of disrupted molecular clockwork on NPC migration. Therefore, we analyzed the impact of Bmal1 deficiency on NPC migration on both the systemic and the cellular level.

## Methods

### Ethics

All animal experiments were approved by the local government, North Rhine-Westphalia State Agency for Nature, Environment and Consumer Protection, Germany (application numbers: 84-02.04.2012.A102, 84-02.04.2014.A314) and in agreement with the ARRIVE international guidelines on the ethical use of animals (Kilkenny et al. 2012). For ethical and economical reasons, sample sizes were kept to a minimum (Festing and Altman 2002).

### Experimental animals

Heterozygous mice with a targeted deletion of Bmal1 (Bmal1^+/−^) on a C57BL/6 background, kindly provided by Christopher Bradfield, were kept for breeding at the local animal facility of University of Düsseldorf to obtain Bmal1^−/−^ and Bmal1^+/+^ littermates. PCR was used to confirm the genotype (Bunger et al. 2000). Mice were housed in standard single cages, with controlled 12 h light/12 h darkness, lights on at 6:00 am and constant temperature conditions. Mice had free access to food and water. And, the mice were killed between 8:00 and 10:00 am.

### In vivo BrdU assay and tissue preparation for immunohistochemistry and immunofluorescence

12 ± 2 weeks male mice (*n* = 8 per genotype) were injected i.p. with BrdU (Roche, Switzerland) at a dose of 100 mg/kg body weight, twice daily at the beginning and at the end of the light phase, on three consecutive days. One group of mice (*n* = 4 mice per genotype) was sacrificed 4 days after the first BrdU administration. The second group of mice (*n* = 4 mice per genotype) was sacrificed 31 days after the first BrdU administration. Animals were anaesthetized using Ketamine/Xylazine (100 mg/10 mg, respectively, /kg body weight), then perfused transcardially with 0.9% NaCl followed by 4% paraformaldehyde using Ministar Peristaltic Pump (World Precision Instruments, USA). Brains were removed from the skull, post fixed in 4% paraformaldehyde for 24 h, and cryoprotected in 20% sucrose for 24 h. Brain hemispheres were sectioned on a cryostat (Leica CM, Germany) into 20-µm-thick sagittal sections.

### Immunohistochemistry and immunofluorescence

Slides were washed with phosphate buffered saline (PBS) with 0.2% Triton-X 100, incubated in 0.6% H_2_O_2_ for 30 min at room temperature (RT), and then rinsed with PBS. For anti-BrdU staining, DNA was denatured by incubation with 2N HCl for 30 min at 37 °C, followed by 0.1 M boric acid for 10 min at RT. Sections were blocked for 1 h with 10% normal goat serum, then incubated overnight at 4 °C with either one or a combination of two of the following primary antibodies against BrdU (1:800, AbD Serotec, UK) 8-hydroxy-2′-deoxyguanosine (8-OH(d)G) (1:250, QED Bioscience, CA, USA), Doublecortin (DCX) (1:1000, Abchem, UK), and glial fibrilary acid protein (GFAP) (1:2000, DAKO, Denmark). For immunohistochemistry, slides were incubated with biotinylated secondary antibody for 3 h at RT, rinsed, then incubated with VECTASTAIN^®^ Elite^®^ ABC solution (Vector Laboratories, CA, USA) for 1 h at RT followed by incubation with 0.05% 3, 3′-Diaminobenzidine tetrahydrochloride hydrate (Sigma-Aldrich, MO, USA) for 5 min. Sections stained against BrdU were counterstained using cresyl violet. Slides were coverslipped using Depex (SERVA Electrophoresis, Germany). For immunofluorescence, slides were incubated with Alexa Fluor 488 goat anti rat IgG (1:500, Thermo Scientific, CA, USA) and Alexa Fluor 568 goat anti rabbit IgG (1:500, Thermo Scientific, CA, USA) for 1 h at RT. Slides were coverslipped using Vectashield Hard Set anti-fade reagent (Vector Laboratories, CA, USA) and kept in darkness at 4 °C.

### Image acquisition and analysis of immunohistochemistry

Images were acquired using BZ-9000E microscope (Keyence, Japan). All samples of an experiment were processed in one session in which microscope and camera settings were kept constant. All analyses were performed by an observer blind to the experimental condition/genotype. DAB-labeled BrdU-immunopositive (+) cells were counted in delineated areas in the SVZ as well as both the proximal and the distal limb of the RMS and in defined layers of the olfactory bulb (Supplementary Fig. 1) using 40x objective in bright field mode. Immunofluorescence was analyzed using respective filters. For analysis of BrdU/DCX co-labeling, 20 randomly selected BrdU^+^ cells in olfactory bulb were examined. Co-labeling was confirmed by 3D reconstruction of Z stack series using BZ Analyser software (Keyence, Japan). The width of RMS, determined by the area stained for DCX, was measured in at least seven different rostrocaudal levels in equivalent sections in each animal. Dispersion of DCX + cells from RMS into the neighboring structure was determined as previously described (Courtes et al. 2011). GFAP immunoreactivity of the glial tube surrounding the RMS, as well as 8-OH(d)G immunoreactivity in RMS and olfactory bulb were quantitatively analyzed using Image J software (http://rsbweb.nih.gov/ij). The threshold of immunoreaction was determined above background in cell body-free neuropil and kept constant for all measurements. The percentage of immunoreactive area relative to the total area was calculated for each section.

### Tissue preparation for gene expression analysis

For gene expression analyses ex vivo, mice (*n* = 9−7 per genotype) were killed by isoflurane and the olfactory bulb was dissected. Total RNA was isolated using RNeasy Lipid Tissue Mini Kit (Qiagen, Germany) according to the manufacturer’s protocol.

### Neurosphere culture

Bmal1^−/−^ mice and their wild-type littermates (Bmal1^+/+^ mice) at age P0 to P3 were decapitated and brains were removed. NPCs were isolated after protocols previously described (Baumann et al. 2014; Fritsche et al. 2011). Briefly, the meninges, the brain stem and the cerebellum were removed. The forebrain was dissected and cut into small pieces (100–150 µm) in ice-cold HBSS and incubated with papain (27 U/ml in HBSS; Worthington PDS Kit) for 10 min at 37 °C. The enzyme was stopped using 1% ovomucoid solution (1 mg/ml in HBSS including Ca^2+^ and Mg^2+^ (Sigma-Aldrich, MO, USA) 1% DNAse I (Roche Diagnostics, Switzerland), 0.5% BSA, 1% ovomucoid stock solution, DMEM. A single cell suspension was received using a sterile sieve (70 µm, Grainer). Cell debris were removed by centrifugation (80×*g*) at 4 °C for 5 min. Cells were seeded into 10 cm tissue culture plates and cultured in proliferation medium (DMEM/F12, B27 supplement, 20 ng/ml EGF, 20 ng/ml bFGF, 1% PenStrep, 10 mM HEPES) in a humidified incubator at 5% CO_2_ for up to 10 days. Neurospheres at a size of 150 µm in diameter were collected and centrifuged at 80×*g* for 10 min at 4 °C. Supernatant was removed and neurospheres were incubated with 1 ml Accutase for 5 min at 37 °C and cultured in a humidified incubator at 5% CO_2_. After four passages, neurospheres reached a diameter of 100–150 µm, single neurospheres were collected and seeded on poly-d-lysine (10 µg/cm^2^)-coated culture plates. For migration assays, neurospheres were cultured in migration medium (DMEM, 1% PenStrep, 10 mM HEPES and 1x B27 without vitamin A) with or without catalase (500 U/ml)(Valdameri et al. 2011), H_2_O_2_ (80 µM) (Perez Estrada et al. 2014) or *N*-acetylcysteine in various concentrations. Cell migration was continuously recorded for 24 h after seeding in cell culture micro-dishes (IBIDI, Germany) using life-cell imaging phase-contrast light microscopy (Axiovert, Zeiss, AxioVision-Software, Zeiss). Data were analyzed in 3 h intervals from 16 neurospheres per individual mouse. The average migration distance was calculated as the distance between the perimeter of the neurosphere and the perimeter of the leading front of the radially migrating cells (Baumann et al. 2015). The average migration velocity in µm/h was calculated based on the migration distance. Migrating cells were identified as neural progenitors by immunocytochemistry for PSA-NCAM (Hack et al. 2002; Hu 2000; Chazal et al. 2000) and DCX (Supplementary Fig. 2). The absence of glia cells was confirmed by GFAP immunoreaction (Supplementary Fig. 2c). Analyses on NPC number (in 16 neurospheres per individual mouse) as well as on filopodia number and length per cell (7 per neurosphere) were performed 24 h after seeding the neurospheres in migration medium using Image J. Means for neurospheres from individual mice were calculated and data were expressed as mean ± SEM of the number of individual mice per group (*n*).

### Cytochemistry and immunocytochemistry

24 h after seeding, cells on culture plates were fixed with 4% PFA for 1 h and rinsed with PBS. For high-affinity F-actin cytochemistry, the cells were incubated with Alexa Fluor 594-conjugated phalloidin (1:30, Thermo Scientific, CA, USA). For immunocytochemistry the cells were incubated with 10% normal goat serum in PBS-T 0.2% at RT for 1 h, followed by an incubation with the primary antibodies against PSA-NCAM (1:200, Cell Signalling Technologies, Danvers, MA, USA), GFAP (1:500, DAKO; Denmark), DCX (1:1000, Abchem, UK), or 8-OH(d)G (1:250, QED Bioscience, CA, USA) for 12 h at RT. Additionally, a fraction of cells on culture plates were treated with 10 µg/µl DNase I (Qiagen, Germany), or 5 µg/µl RNase (Qiagen, Germany), before incubation with 8-OH(d)G antibody. Culture plates were rinsed in PBS-T 0.2%, followed by incubation with Alexa Fluor 488 or Alexa Fluor 647 conjugated secondary antibody against rabbit IgG (1:500, Molecular Probes, USA) for 1 h at RT. Cell nuclei were counterstained with NucBlue (Thermo Scientific, CA, USA). Culture plates were coverslipped using Vectashield Hard Set anti-fade reagent (Vector Laboratories, CA, USA) and stored in darkness at 4 °C. The integrated density of 8-OH(d)G-Ir in NPCs above cell-free background was analyzed using Image J software.

### Immunoblot

24 h after seeding, NPCs (from 16 neurospheres per mouse) were lysated in M-Per buffer (Thermo Scientific, Germany) and sonicated on ice. Protein concentration was determined using BCA kit (Thermo Scientific, CA, USA). PAGE and Western blotting was performed using XCell Sure Lock Blot module (Thermo Scientific, CA, USA) following manufacturer’s instructions. The membranes were washed in TBST and incubated with blocking solution (TBST containing 5% milk powder, fat free, Sucofin, Germany) for 1 h at room temperature. Membranes were incubated with anti β-actin (1:1000, Cytoskeleton, CO, USA) and with anti-catalase (1:2500, Santa Cruz, clone H-9: sc271803, Germany), anti phospho-cofilin (1:1000, Cell Signalling Technologies, MA, USA), or anti-SOD2 (1:1000, Abcam, UK) in 3% BSA in TBST for 12 h at 4 °C. After washing, membranes were incubated with secondary HRP-conjugated antibody for 1 h at RT. After washing, immunoreactive bands were visualized using Immobilon Western Chemiluminescent HRP substrate (Millipore, Germany) on the Molecular Imager® ChemiDoc™XRS (BioRad, CAa, USA). Immunoreactive bands were normalized against ß-actin using densitometric analyses with Molecular Imager® ChemiDoc™XRS software (BioRad, CA, USA). Catalase and SOD2 (+β-actin) immunoblot were performed three times with NPCs from different mice (*n* = 5 mice per genotype). p-Cofilin (+β-actin) immunoblot was performed once with NPCs from different mice (*n* = 3 mice per genotype).

### ROS imaging

24 h after seeding, oxidative stress in NPCs was detected using the ROS-sensitive dye CellROX® (Thermo Scientific, CA, USA) kit. NPCs were incubated with CellROX® reagent (5 µM) for 30 min at 37 °C. As a positive control, NPCs were incubated with 1 mM tert-butylhydroperoxid (TBHP) for 60 min at 37 °C prior CellROX® assay. Medium was removed, and NPCs were fixed in 4% paraformaldehyde for 15 min at 37 °C. The number of fluorescent cells above cell free background was counted using Image J.

#### Immunoprecipitation of oxidized RNA

Immunoprecipitation of oxidized RNA was performed as previously described (Shan et al. 2003; Gorg et al. 2008). Briefly, 24 h after seeding, NSCs were lysated in buffer containing 10 mM Tris, pH8, 10 mM NaCl_2_, 1% Triton X 100 (Merck, Germany), 1.33 mg/ml Proteinase K (Sigma-Aldrich, MO, USA), 400 U/ml DNase I and 1 U/ml RNase Inhibitor (Qiagen, Germany) for 60 min at 37 °C. After centrifugation, the supernatant was collected and frozen at − 20 °C. 8-OH(d)G antibody (1:200, clone 15A3, Acris, AM03160PU-N, Germany) which was crosslinked to G sepahrose beads (Abcam, UK) using DMP (dimethyl pimelimidate, Abcam, UK) according to respective manufacturers protocols. The immunobeads were incubated with cell lysates for 2 h at 4 °C. Immunopurified RNA was obtained by centrifugation for 30 s at 14,900 *g*, extraction with phenol/chloroform and precipitation with 100% isopropanol.

### Gene expression analyses

cDNA of total RNA or immunoprecipitated oxidized RNA was prepared using QuantiTect Reverse transcription kit (Qiagen, Germany). The following PCR program was used for amplification: 15 min at 42 °C, 1 min at 95 °C then at 4 °C. For PCR, Applied Biosystems StepOne™ Real-Time PCR Systems was used. Based on SYBR green reagents (KAPA SYBR FAST qPCR Kit master mix ABI Prism (KAPA Biosystems, South Africa) and the following primer sequences were used:

*Aldh2* (F: TGCTACGATGTGTTTGGGGC, R: TTCACTTCTGTGTACGCCTGC), *Nqo1* (F: CATTGCAGTGGTTTGGGGTG, R: TCTGGAAAGGACCGTTGTCG), *Hgf* (F: TGATCCCCCATGAACACAGC, R: CCCCTCGAGGATTTCGACAG), *Prdm16* (F: GCCCCATGATGGACAAGACA, R: TCCCAGGATGAGGTCTGGAG), *Sod2* (F: TCCGTCCGTCGGCTTCTCGT, R: TCACCGCTTGCCTTCTGCTCG), *Pparα* (F: TGAGGAAGCCGTTCTGTGAC; R: GTTTAGAAGGCCAGGCCGAT), *Cat* (F: GCCAATGGCAATTACCCGTC, R: GAGGCCAAACCTTGGTCAGA), 18srRNA (F: TACCGCCCCTCGTAGACAC, R: GCTCTGACCTCGCCACC), *actin* ß (*Actb*) (F: CCTTCCAGCAGATGTGGATCA, R: CTAGAAGCACTTGCGGTGCA), *Gapdh* (F: TGC CAA GGC TGT GGG CAA GG, R: CCA GGC GGC ACG TCA GAT CC). *Actb* and *Gaphd* were used as housekeeping genes. The average of the Ct values of both housekeeping genes was used to calculate the relative expression levels for the respective genes of interest (Vandesompele et al. 2002).

### Statistical analysis

Statistics were calculated using Graph Pad Prism 6 software. Values are expressed as mean ± SEM. Differences between two genotypes or between two treatments were analyzed by Mann–Whitney *U* Test. Values were considered significantly different with *P* < 0.05.

### Data availability

The datasets generated during and/or analyzed during the current study are available from the corresponding author on reasonable request.

## Results

### Bmal1 deficiency affects NPC proliferation in the SVZ and RMS

**Fig. 1:**
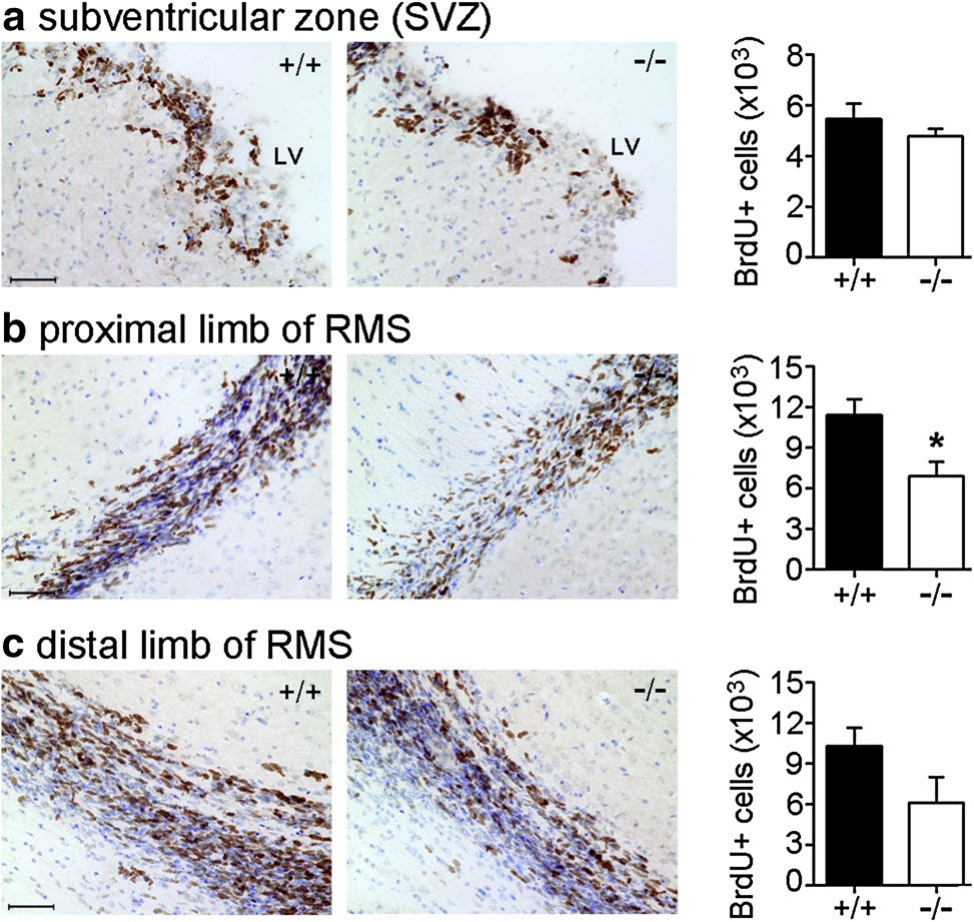
Bmal1 deficiency affects NPC proliferation. Representative photomicrographs of BrdU immunoreaction (brown precipitate) and quantification of BrdU-immunopositive cells in the subventricular zone (SVZ) and rostral migratory stream (RMS) in Bmal1^+/+^ mice (+/+) and Bmal1^−/−^ mice (-/-) four days after the first day of BrdU injection. Counterstaining with cresyl violet (blue) was used to highlight the anatomical locations. **a** Subventricular zone (SVZ), *LV* lateral ventricle **b** proximal limb of RMS, *P < 0.05. **c** Distal limb of (RMS). *n* = 4 mice per genotype. Scale bars = 50 µm

To assess proliferation in the SVZ and RMS, the number of bromodeoxyuridine positive (BrdU^+^) cells was analyzed 4 days after the first BrdU injection. The number of BrdU^+^ cells in the SVZ (Fig. 1a) as well as in the proximal (Fig. 1b) and in the distal RMS (Fig. 1c) was reduced in Bmal1^−/−^ mice as compared to Bmal1^+/+^ mice. The number of BrdU^+^cells was significantly different in the proximal RMS between Bmal1^+/+^ mice and Bmal1^−/−^ mice (*P* = 0.028, *n* = 4 per genotype) (Fig. 1b). This indicates an effect of Bmal1 deficiency on progenitor cell proliferation in Bmal1^−/−^ mice.

### Bmal1 deficiency affects migration of NPCs to the olfactory bulb

**Fig. 2:**
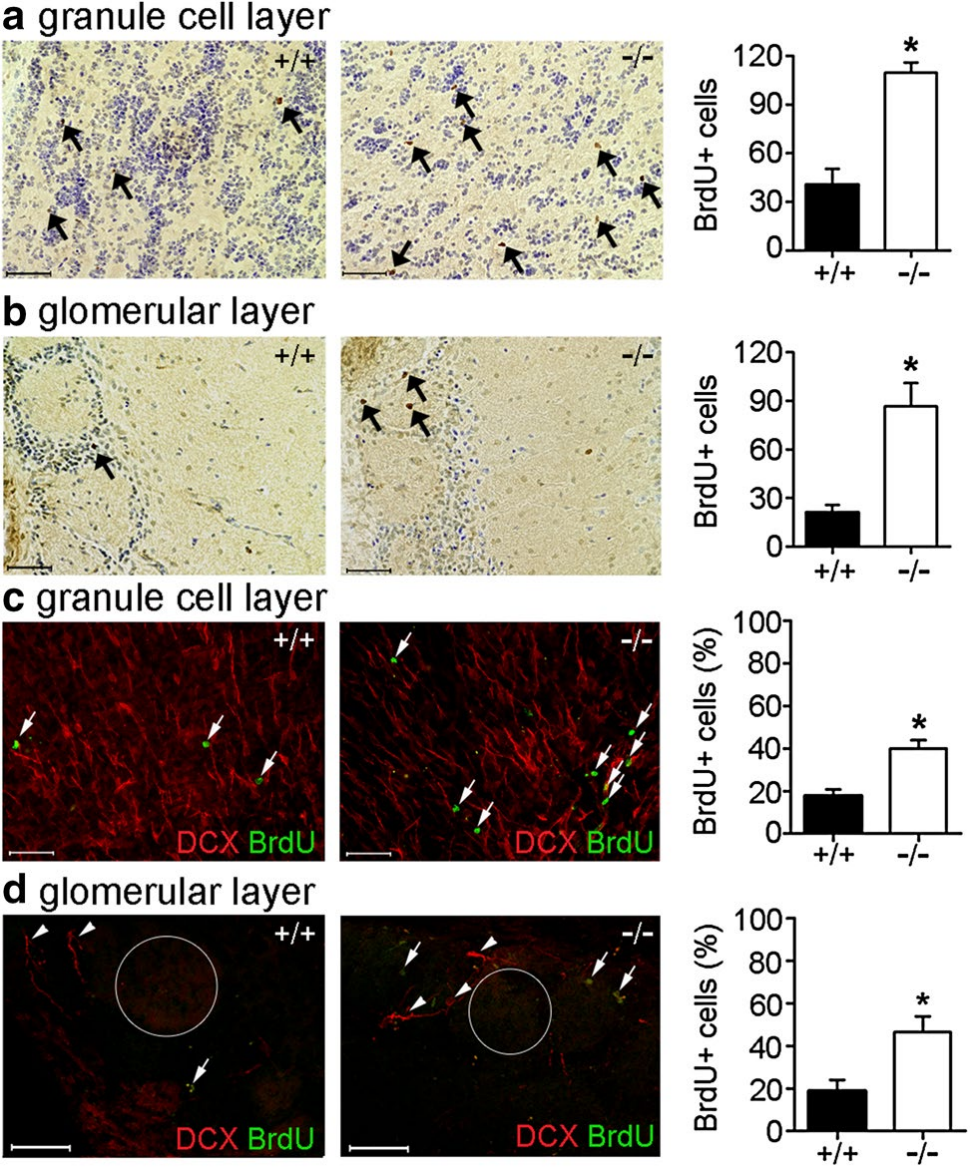
Bmal1 deficiency affects the number of newly developed neurons reaching the olfactory bulb within 4 days. Representative photomicrographs and quantification of BrdU-immunopositive cells (arrows) in the olfactory bulb of Bmal1^+/+^ mice (+/+) and Bmal1^−/−^ mice (-/-). BrdU immunoreaction (brown precipitate) and cresyl violet staining (blue) in (**a**) granule cell layer (**b**) glomerular layer. Immunofluorescence for BrdU and DCX, as a marker of newly developed neurons, and quantification of DCX^+^/BrdU^+^ cell as percentage of total BrdU^+^ cells in (**c**) granule cell layer, (**d**) glomerular layer, arrowheads indicate DCX^+^ cells, glomeruli are indicated by circles. *P < 0.05, *n* = 4 mice per genotype. Scale bars = 50 µm

Four days after the first injection with BrdU, the number of BrdU^+^ cells reaching the olfactory bulb was significantly higher in Bmal1^−/−^ mice as compared to Bmal1^+/+^ mice in both, the granule cell layer (*P* = 0.028, *n* = 4 mice per genotype) (Fig. 2a) and the glomerular layer (*P* = 0.028, *n* = 4 mice per genotype) (Fig. 2b). In the glomerular layer, the BrdU^+^ cells were present as periglomerular cells, between the glomeruli (Fig. 2b, d). DCX, as a marker for neuronally determined NPCs, was present in the granule cell layer and also periglomerular (Fig. 2d). The percentage of cells co-labeled with BrdU and DCX was significantly higher in Bmal1^−/−^ mice as compared to Bmal1^+/+^ mice in both, the granule cell layer (*P* = 0.028, *n* = 4 mice per genotype) (Fig. 2c) and the glomerular layer (*P* = 0.028, *n* = 4 mice per genotype) (Fig. 2d) of the olfactory bulb. This indicates that within 4 days more newborn neurons reach the olfactory bulb in Bmal1^−/−^ mice as compared to Bmal1^+/+^ mice.

However, 31 days after the first injection with BrdU, the number of BrdU^+^ cells in the olfactory bulb was not different between Bmal1^+/+^ mice and Bmal1^−/−^ mice (Supplementary Fig. 3). This suggests that in Bmal1^+/+^ mice, newborn neurons migrate faster towards the olfactory bulb.

### Bmal1 deficiency affects formation of the glial tube surrounding the RMS and is associated with increased oxidative stress

**Fig. 3:**
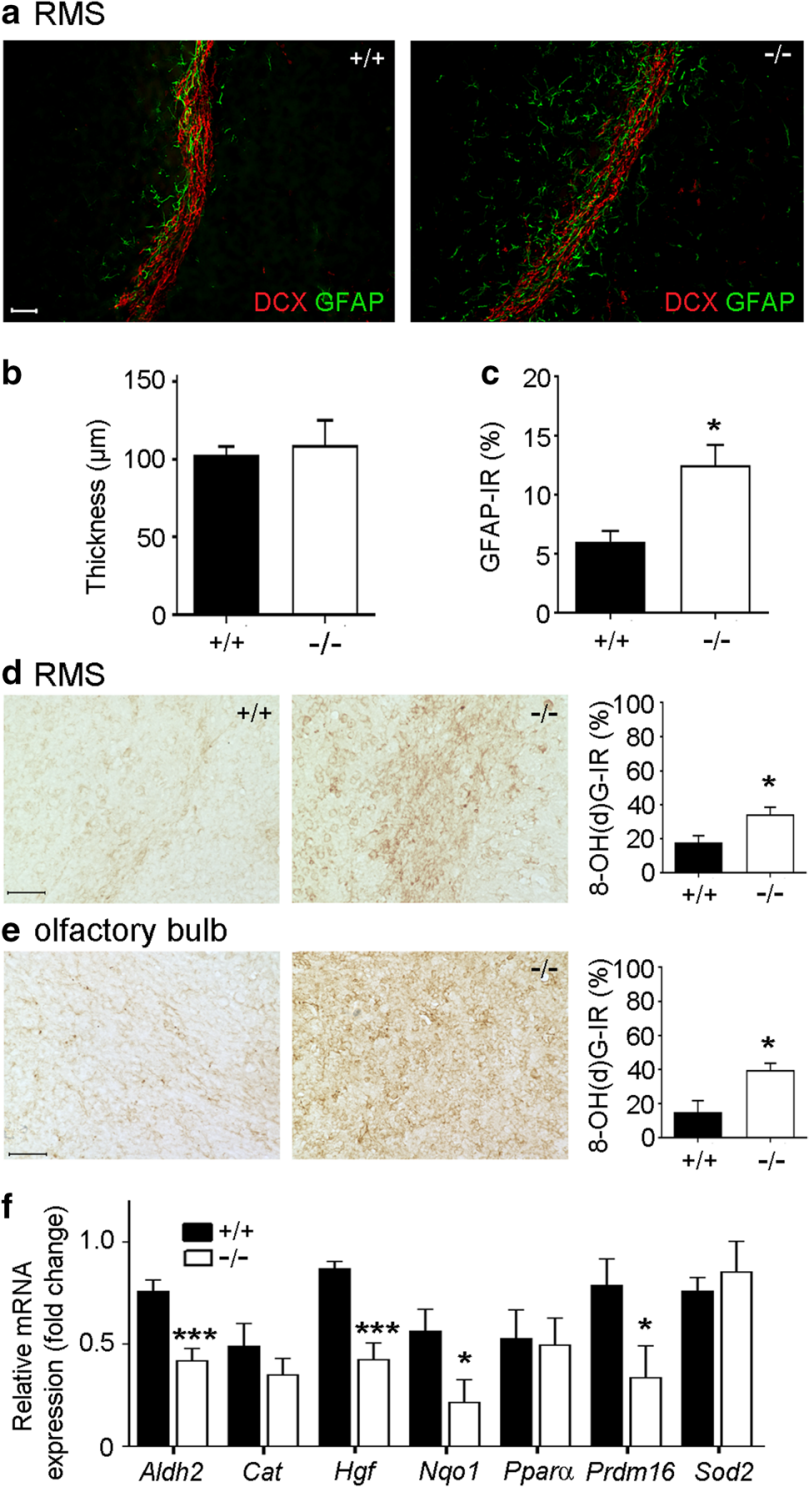
Bmal1 deficiency affects formation of the glial tube surrounding the RMS, oxidative stress and ROS defense gene expression. **a** Representative photomicrographs show DCX^+^ migrating neuroblasts forming the RMS surrounded by GFAP^+^ glia cells in Bmal1^+/+^ mice (+/+) and Bmal1^−/−^ mice (−^/^−). Scale bar = 50 µm. **b** Quantification of RMS thickness, determined by DCX immunoreaction (IR), *n* = 4 per genotype **c** quantification of glial tube surrounding the RMS, determined by GFAP-IR, *P < 0.05, n = 4 per genotype. **d** Representative photomicrographs and quantification of 8-OH(d)G -IR in RMS of Bmal1^+/+^ mice (*n* = 4) and Bmal1^−/−^ mice (*n* = 5), *P < 0.05. Scale bar = 50 µm. **e** Representative photomicrographs and quantification of 8-OH(d)G-IR in the olfactory bulb of Bmal1^+/+^ mice (*n* = 4) and Bmal1^−/−^ mice (*n* = 5), *P < 0.05. Scale bar = 50 µm. **f** Quantification of relative ROS defense gene expression in Bmal1^+/+^ mice (black bars, *n* = 9) and Bmal1^−/−^ mice (*n* = 7, white bars), *P < 0.05, ***P < 0.001

The thickness of RMS, determined by the DCX^+^ area, was not different between Bmal1^+/+^ mice and Bmal1^−/−^ mice (Fig. 3a, b). However, the percentage of GFAP^+^ area surrounding the RMS was higher in Bmal1^−/−^ mice as compared to Bmal1^+/+^ mice (*P* = 0.028, *n* = 4 mice per genotype) (Fig. 3a, c). This suggests an enhanced formation of the glial tube surrounding the RMS in Bmal1^−/−^ mice. This was associated with a significantly higher cytoplasmic immunoreactivity of the marker for oxidative stress, 8-hydroxy-2′-deoxyguanosine (8-OH(d)G), in Bmal1^−/−^ mice (*n* = 5) as compared to Bmal1^+/+^ mice (*n* = 4) in the RMS (*P* = 0.0317, Fig. 3d) and in the olfactory bulb (*P* = 0.0159, Fig. 3e). Consistently, expression levels of genes involved in detoxification of ROS such as *Aldh2* (*P* = 0.0007), *Hgf* (*P* = 0.0002), Nqo1 (*P* = 0.033), and *Prdm16* (*P* = 0.04) were down-regulated in the olfactory bulb of Bmal1^−/−^ mice (*n* = 7) as compared to Bmal1^+/+^ mice (*n* = 9) (Fig. 3f). These data suggest an increased recruitment of astrocytes to the RMS associated with high oxidative stress in Bmal1^−/−^ mice.

### Bmal1 deficiency affects migration of NPCs, filopodia morphology and phospho-cofilin expression in vitro

**Fig. 4:**
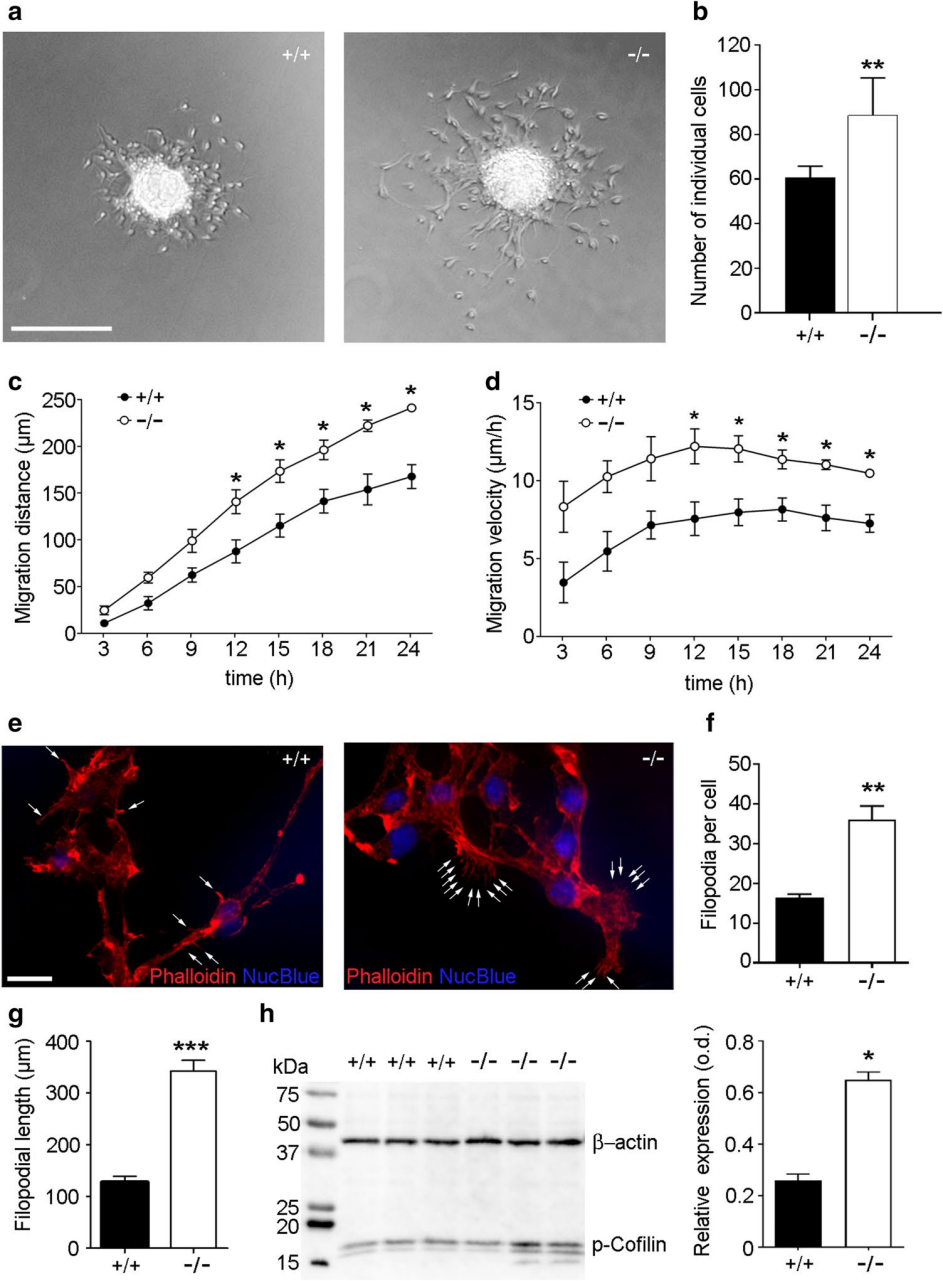
Bmal1 deficiency affects migration of NPCs, filopodia morphology and phospho-cofilin expression in vitro. **a** Representative microphotographs of neurospheres and out-migrating NPCs derived from Bmal1^+/+^ mice (+/+) and Bmal1^−/−^ mice (−/−). Scale bar = 300 µm. **b** Quantification of detached NPCs 24 h after seeding. The number of detached NPCs was significantly higher in Bmal1^−/−^ as compared to Bmal1^+/+^, *P < 0.05, *n* = 6 mice per genotype. **c** Migration distance and consequently (**d**) migration velocity were significantly different between Bmal1^+/+^ and Bmal1^−/−^ during the first 24 h after seeding *P < 0.05, *n* = 4 mice per genotype. **e** Representative photomicrographs of neuroblasts 24 h after seeding with F-actin staining (Phalloidin) and nuclei staining (NucBlue), scale bar 20 µm. Arrows indicate filopodia. **f** Quantification of filopodia number per cell, **P < 0.01, *n* = 6 mice per genotype (**g**) Quantification of filopodial length, **P < 0.01, *n* = 6 mice per genotype **h** representative immunoblots and quantification of phospho (p)-cofilin in NPCs from Bmal1^+/+^ mice and Bmal1^−/−^ mice. *P < 0.05, *n* = 4 mice per genotype

The intrinsic effect of Bmal1 deficiency on NPC migration was analyzed in neurosphere cultures derived from Bmal1^+/+^ mice (Fig. 4a, supplemental video 1) and Bmal1^−/−^ mice (Fig. 4a, supplemental video 2). 24 h after seeding, the number of individual cells which migrated out of the neurosphere was higher in Bmal1^+/+^ as compared to Bmal1^−/−^ (*P* = 0.0022, *n* = 6 mice per genotype) (Fig. 4b). Moreover, migration distance was significantly higher in NPCs from Bmal1^−/−^ mice as compared to NPCs from Bmal1^+/+^ mice from 12 to 24 h after seeding (*P* = 0.0286; *n* = 4 mice per genotype) (Fig. 4c). Consequently, migration velocity was also significantly higher in NPCs from Bmal1^−/−^ mice as compared to NPCs from Bmal1^+/+^ mice from 12 to 24 h after seeding (*P* = 0.0286; *n* = 4 mice per genotype) (Fig. 4d). This indicates an intrinsic promoting effect of Bmal1 deficiency on cell detachment and migration. Morphologically, filopodia were more frequent (*P* = 0.0022, *n* = 6 mice per genotype) (Fig. 4e, f) and longer (*P* = 0.0022, *n* = 6 mice per genotype) (Fig. 4e, g) in NPCs from Bmal1^−/−^ mice as compared to NPCs from Bmal1^+/+^ mice, consistent with enhanced cell motility. Moreover, in NPCs from Bmal1^−/−^ mice the level of phospho-cofilin, the ROS-sensitive mediator of actin dynamics (Bernstein and Bamburg 2010), was significantly higher as compared to NPCs from Bmal1^+/+^ mice (*P* = 0.0286, *n* = 4 mice per genotype) (Fig. 4h), consistent with cytoskeleton stabilization.

### Bmal1 deficiency affects ROS production, expression of oxidative stress-related genes and RNA oxidation in NPCs in vitro

**Fig. 5:**
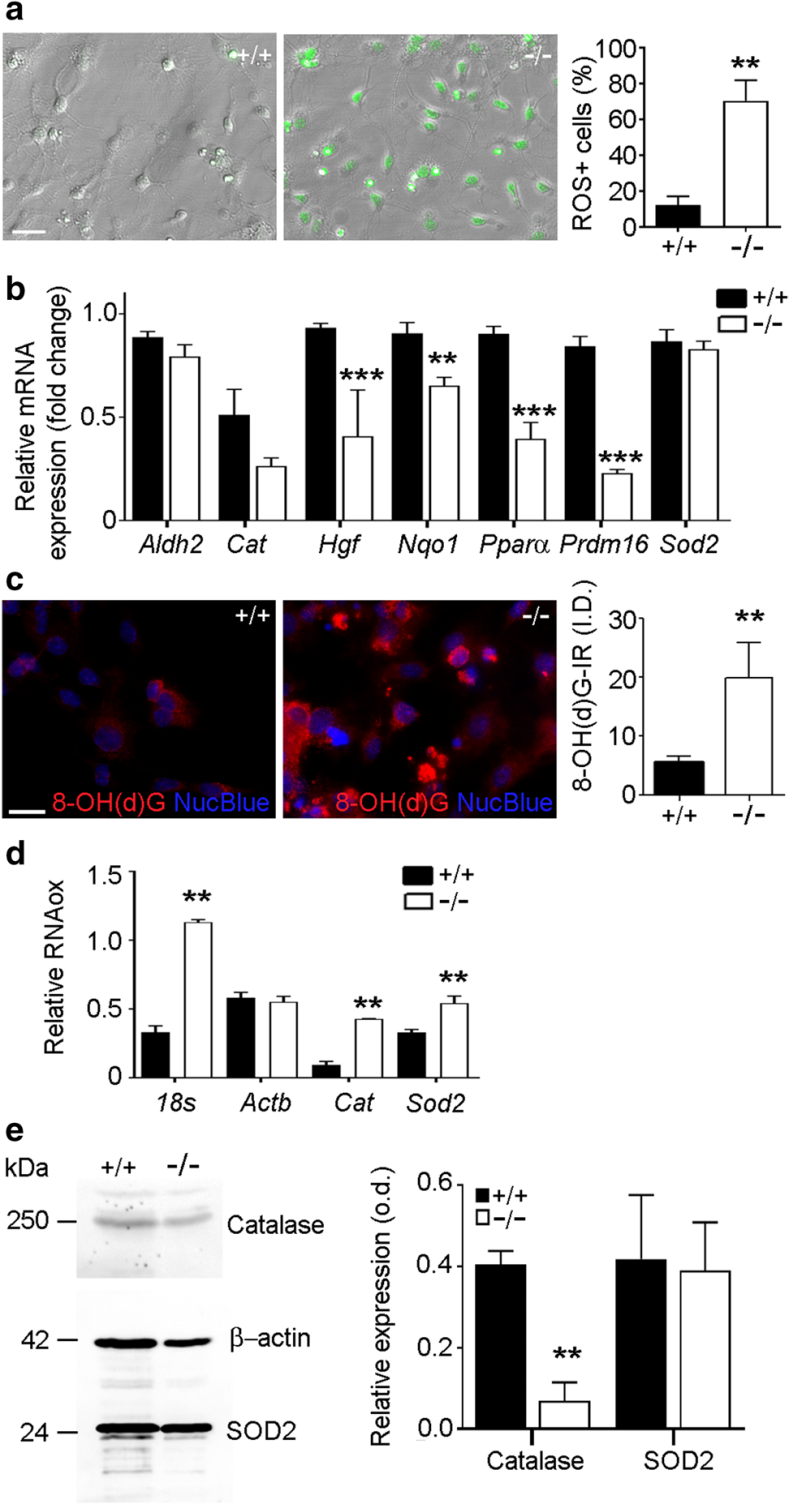
Bmal1 deficiency is associated with high ROS production, reduced ROS defense gene expression, increased RNA oxidation and decreased catalase levels in NPCs. **a** Representative photomicrographs and quantification of NPCs derived from Bmal1^+/+^ mice (+/+) and Bmal1^−/−^ mice (−/−) with ROS-sensitive dye CellROX® 24 h after seeding, **P < 0.001, *n* = 6 mice per genotype. Scale bar = 30 µm (**b**) quantification of relative ROS defense gene expression in Bmal1^+/+^ mice and Bmal1^−/−^ mice, n = 9 mice per genotype. **P < 0.01; ***P < 0.0001. **c** Representative photomicrographs and quantification of 8-OH(d)G immunoreaction (IR, red) in NPCs as integrated density (I.D.), **P < 0.01, *n* = 5 mice per genotype. Scale bar = 20 µm **d** quantification of oxidized RNA (RNAox) levels after immunopurification from total RNA with 8-OH(d)G antibody. **P < 0.001, *n* = 6 mice per genotype. **e** Representative immunoblots and quantification of relative catalase and SOD2. **P < 0.01, *n* = 6 mice per genotype

NPCs derived from Bmal1^−/−^ mice showed a significant increase in the number of cells positive for ROS-sensitive Cell ROX® as compared to NPCs derived from Bmal1^+/+^ mice (*P* = 0.0022, *n* = 6 mice per genotype) (Fig. 5a). Consistently, expression levels of genes involved in ROS detoxification such as *Hgf, P* < 0.0001; *Nqo1, P* = 0.0039; *Pparα, P* < 0.0001; and *Prdm, P* < 0.0001 were significantly down-regulated in NPCs from Bmal1^−/−^ mice as compared to NPCs from Bmal1^+/+^ mice (*n* = 9 mice per genotype) (Fig. 5b). However, in contrast to the in vivo situation *Aldh2* was not down-regulated in NPCs from Bmal1^−/−^ mice. This suggests a cell type-specific expression of ROS detoxification genes. NPCs derived from Bmal1^−/−^ mice showed significantly higher cytoplasmic 8-OH(d)G-Ir as compared to NPCs derived from Bmal1^+/+^ mice (*P* = 0.0079, *n* = 5 mice per genotype) (Fig. 5c). Cytoplasmic 8-OH(d)G -Ir could not be prevented by pretreatment of NPCs with DNAse but by pretreatment with RNAse (Supplementary Fig. 4), indicating that 8-OH(d)G-Ir represents oxidized RNA. Oxidized RNA was immunopurified from total RNA using an 8-OH(d)G antibody and analyzed by real-time PCR for oxidized 18 s-rRNA as a positive control (Gorg et al. 2008), oxidized mRNAs encoding for *β* -*actin* as our house keeper for real-time PCR and immunoblot which has been shown to be unaffected by oxidation in similar conditions (Shan et al. 2003), or for *catalase* (*Cat)* and *Sod2* as the genes of interest. In NPCs derived from Bmal1^−/−^ mice, the levels of oxidized *18 s* (*P* = 0.0022), oxidized *Cat* (*P* = 0.0022) and *Sod2* (*P* = 0.002) mRNA were significantly elevated as compared to NPCs derived from Bmal1^+/+^ mice (*n* = 6 mice per genotype) (Fig. 5d). As RNA oxidation is associated with insufficient translation into protein (Shan et al. 2003), we analyzed catalase and SOD2 protein levels. In NPCs from Bmal1^−/−^ mice, catalase protein level was significantly lower as compared to NPCs from Bmal1^+/+^ mice (*P* = 0.0087, *n* = 6 mice per genotype) (Fig. 5e). Thus, a higher level of oxidized *Cat* mRNA is associated with lower catalase protein levels. In contrast, the protein level of SOD2 was not affected by Bmal1 deficiency (Fig. 5e). This suggests a different dynamic in mRNA translation and/or protein stability between catalase and SOD2.

### Bmal1 deficiency affects Catalase protein expression and catalase treatment restores wild-type NPC migration phenotype

**Fig. 6:**
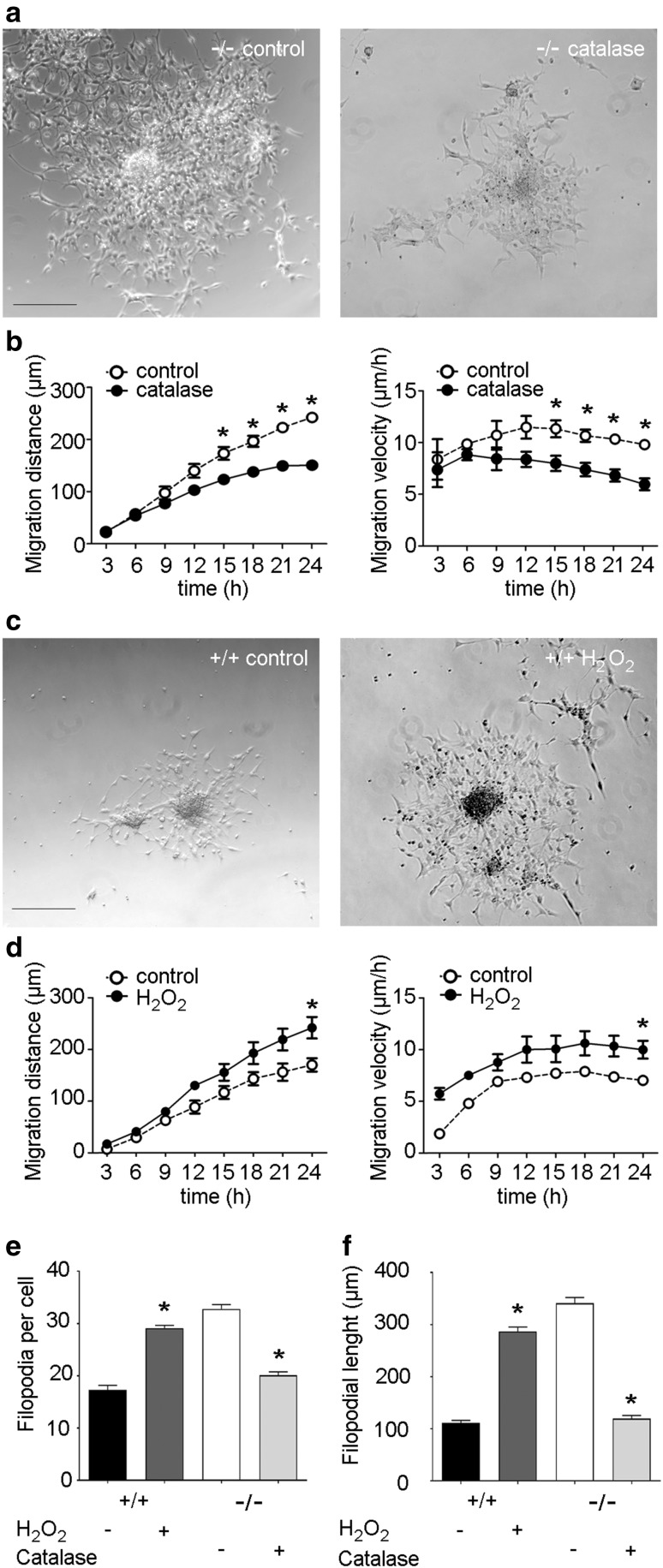
Treatment with catalase restores wild-type migration phenotype, treatment with hydrogen peroxide mimics Bmal1-deficient migration phenotype. **a** Representative photomicrographs of NPCs from Bmal1^−/−^ mice (−/−) treated with vehicle (control) or 500 U/ml catalase (catalase) for 24 h. Scale bar: 200 µm. **b** Time course of migration distance and velocity after treatment of NPCs from Bmal1^−/−^ mice with vehicle (control) or 500 U/ml catalase (catalase) during the first 24 h after seeding. *P < 0.05, n = 4 mice per group. **c** Representative photomicrographs of NPCs from Bmal1^+/+^ mice (+/+) treated with vehicle (control) or 80 µM hydrogen peroxide (H_2_O_2_) for 24 h. Scale bar: 200 µm. **d** Time course of migration distance and velocity after treatment of NPCs from Bmal1^+/+^ mice with vehicle (control) or 80 µM H_2_O_2_ (H_2_O_2_) during the first 24 h after seeding. *P < 0.05, *n* = 4 mice per group. **e** Quantification of filopodia number in NPCs from Bmal1^+/+^ mice treated with (+) or without (−) H_2_O_2_ or in NPCs from Bmal1^−/−^ mice treated with (+) or without (−) catalase. *P < 0.05, *n* = 4 mice per genotype. **f** Quantification of filopodia length in NPCs from Bmal1^+/+^ mice treated with (+) or without (−) H_2_O_2_ or in NPCs from Bmal1^−/−^ mice treated with (+) or without (−) catalase. *P < 0.05, *n* = 4 mice per group

Next, we tested whether there is a causal relationship between down-regulated catalase levels and enhanced NPC migration. Treatment of NPCs from Bmal1^−/−^ mice with catalase resulted in a significant decrease in migration distance and velocity from 15 to 24 h after seeding (*P* = 0.0286; *n* = 4 mice per group) as compared to vehicle treatment (Fig. 6a, b). This is reminiscent of migration distance and velocity of vehicle-treated NPCs from Bmal1^+/+^ mice (Fig. 6c, d). This indicates a direct causal relationship between catalase and NPC migration. As reduced catalase capacity results in accumulation of hydrogen peroxide (H_2_O_2_), we tested whether there is a causal relationship between H_2_O_2_ and NPC migration. Indeed, treatment of NPCs from Bmal1^+/+^ mice with 80 µM H_2_O_2_ resulted in a significant increase in migration distance and velocity 24 h after seeding (*P* = 0.0286; *n* = 4 mice per group) as compared to vehicle treatment (Fig. 6c, d). This is reminiscent of migration distance and velocity of vehicle-treated NPCs from Bmal1^−/−^ mice and indicates a direct causal relationship between H_2_O_2_ and NPC migration. Consistently, the number (Fig. 6e) and length (Fig. 6f) of filopodia was significantly higher in NPCs from Bmal1^+/+^ mice treated with hydrogen peroxide as compared to vehicle-treated NPCs from Bmal1^+/+^ mice (*P* = 0.0286, *n* = 4 mice per group). Similarly, the number (Fig. 6e) and length (Fig. 6f) of filopodia was significantly lower in NPCs from Bmal1^−/−^ mice treated with catalase as compared to vehicle-treated NPCs from Bmal1^−/−^ mice (*P* = 0.0286, *n* = 4 mice per group).

In contrast, treatment of NPCs from Bmal1^−/−^ mice with *N*-acetylcysteine, which serves as a precursor for the antioxidant glutathione, did not affect NPC migration distance and velocity (Supplementary Fig. 5).

## Discussion

The SVZ with RMS represents the most extensive germinal niche and a source for long-range neuronal migration in the adult mammalian brain (Lim and Alvarez-Buylla 2016). Here we show for the first time, that proliferation and migration of NPCs in this neurogenic niche is affected in mice with a targeted deletion of the core clock gene *Bmal1*. Our in vitro studies confirm an increase in migration velocity in NPCs derived from Bmal1^−/−^ mice as a consequence of reduced catalase activity and high oxidative stress. Thus, this study provides novel evidence for the role of Bmal1 in regulating NPC migration.

The SVZ (Luskin 1993; Merkle et al. 2004) and RMS (Gritti et al. 2002), give rise to NPCs migrating to the olfactory bulb. Proliferation of NPCs was reduced in Bmal1^−/−^ mice especially in the proximal limb of RMS of the SVZ. This is consistent with our previous observation that Bmal1 deficiency is associated with reduced proliferation of type2b NPCs in the SGZ of the hippocampus (Ali et al. 2015). However, despite this reduction in proliferation, we found a significantly higher number of BrdU^+^/DCX^+^, and thus neuronally determined, NPCs in the olfactory bulb of Bmal1^−/−^ mice 4 days after the first BrdU injection. This indicates an accelerated migration of NPCs from Bmal1^−/−^ mice to their target region. This is consistent with our previous observation of a higher fraction of newborn cells in the outer two-thirds of the dentate gyrus in Bmal1^−/−^ mice also indicating enhanced migration (Ali et al. 2015).

In the olfactory bulb, the glomerular layer is the initial site for synaptic processing of odor information. A glomerulus is made up of a globular tangle of axons from the olfactory receptor neurons, and dendrites from the mitral and tufted cells, as well as from cells that surround the glomerulus such as the periglomerular interneurons (Mori et al. 2006). The granular cell layer consists mainly of interneurons that receive input from mitral cells and inhibit their firing by lateral inhibition (Scott et al. 1993). Most of the adult-born neurons, which migrate to the olfactory bulb, target both the glomerular layer and granule cell layer where they mature into interneurons and integrate into the neuronal network (Whitman and Greer 2007). We found a higher number of neuronally determined NPCs in both the glomerular and the granule cell layer. Thus, the effect of Bmal1 deficiency on NPC migration was not exclusive to a subregion of their target in the olfactory bulb.

However, despite an initially higher number of newborn cells in the olfactory bulb of Bmal1^−/−^ mice, the same number of proliferating cells reaches the olfactory bulb in Bmal1^−/−^ mice and Bmal1^+/+^ mice after 31 days. This indicates, that the same number of “slower” Bmal1^+/+^ NPCs and “faster” Bmal1^−/−^ NPCs reach the olfactory bulb eventually. Thus, we do not expect a difference in size or function of the olfactory bulb between the two genotypes. Newly generated neurons in the olfactory bulb are important for odor discrimination (Gheusi et al. 2000; Imayoshi et al. 2008). In Bmal1^−/−^ mice, the circadian rhythm of odor discrimination is abolished, but the overall odor discrimination sensitivity is not affected (Granados-Fuentes et al. 2011). Thus, Bmal1 deficiency does not seem to affect integration of adult-born NPCs into neuronal circuits of the olfactory bulb but only migration velocity.

Extrinsic and intrinsic factors modulate migration of NPCs from the SVZ to the olfactory bulb (Murase and Horwitz 2004). The astrocytic tube surrounding the RMS synthesizes and secretes different growth factors which are crucial for migration; such as vascular endothelial growth factor (VEGF) (Bozoyan et al. 2012), and glial cell-derived neurotrophic factor (GDNF) (Paratcha et al. 2006). Furthermore, the interaction of migrating neuroblasts and the surrounding astrocytes via GABA modulates the migration speed (Ma et al. 2005). In Bmal1^−/−^ mice, we found an enhanced formation of glial tube surrounding the migrating neuroblasts. This might facilitate the migration of neuroblasts towards their target regions in the olfactory bulb. The enhanced formation of glial tube may be attributed to generalized astrogliosis reported in the brain of Bmal1^−/−^ mice as a consequence of increased oxidative stress and neurodegeneration (Musiek et al. 2013; Kondratova et al. 2010).

Imbalance of the redox homeostasis in Bmal1-deficient mice has been implicated in accelerated aging, neurodegeneration, cognitive deficits and impaired adult neurogenesis (Musiek et al. 2013; Kondratova et al. 2010; Ali et al. 2015). Cellular oxidative stress reflects an imbalance between the production of reactive oxygen species and the ability to detoxify free radicals that damage all components of the cell, including proteins, lipids, RNA and DNA. Here we show an increase in nucleotide oxidation in the olfactory bulb of Bmal1^−/−^ suggesting a higher risk for DNA and RNA damage. We analyzed a variety of genes involved in cerebral redox homeostasis. NQO1 belongs to the NAD(P)H dehydrogenase (quinone) family and is important for cellular antioxidant defense. Aldh2 belongs to the aldehyde dehydrogenase family of enzymes that catalyze the chemical transformation from acetaldehyde to acetic acid hand functions as a protector against oxidative stress (Ohta et al. 2004). *Nqo1* and *Aldh2* are rhythmically expressed with increasing levels during the light phase and dramatically down-regulated in the cerebral cortex of brain-specific Bmal1-deficient mice (Musiek et al. 2013). Consistently, we found a down-regulation of *Nqo1* and *Aldh2* in the olfactory bulb of Bmal1^−/−^ sacrificed during the light phase. *Prdm16* encodes a transcription factor which regulates the cellular redox status and the expression of *Hgf* which encodes for an important growth factor in stem cells including NPCs (Chuikov et al. 2010). PPARs (peroxisome-proliferator-activated receptors) are a superfamily of nuclear receptor that regulate the expression of genes associated with lipid metabolism and play an important role for the pathogenesis of neurodegeneration (Heneka and Landreth 2007). *Pparα* is a target gene of BMAL1 and CLOCK (Oishi et al. 2005) and PARα agonists upregulate the activity of the enzyme catalase which catalyzes the decomposition of hydrogen peroxide to water and oxygen. *Prdm16, Hgf*, and *Pparα* were significantly down-regulated in the olfactory bulb of Bmal1^−/−^ mice. These data emphasize the important role of BMAL1 in the regulation of genes involved in both the production and the detoxification of reactive oxygen species in the brain.

To exclude systemic effects, we further analyzed NPC migration in vitro. Consistent with our in vivo data, NPCs derived from Bmal1^−/−^ mice showed a higher dispersal into individual cells and migrated for longer distance and faster as compared to NPCs derived from Bmal1^+/+^ mice. This indicates an intrinsic, cell autonomous, positive effect of Bmal1 deficiency on NPC detachment and motility. Consistently, NPCs from Bmal1^−/−^ mice showed more and larger filopodia. This is in agreement with the molecular clockwork affecting actin dynamics (Gerber et al. 2013). Moreover, NPCs from Bmal1^−/−^ mice showed a higher level of phospho-cofilin, the ROS-sensitive mediator of actin dynamics (Bernstein and Bamburg 2010). NPCs from Bmal1^−/−^ mice showed increased ROS, equally to increased ROS levels in the brain (Kondratova et al. 2010). Consequently, this was associated with a dysregulation of redox defense genes. Although, in contrast to the in vivo situation, *Aldh2* expression was not down-regulated in Bmal1^−/−^-NPCs, suggesting a systemic and/or a cell type-specific effect. However, *Prdm16* encoding for a transcription factor which regulates the redox status and the expression of *Hgf* in stem cells including NPCs (Chuikov et al. 2010) and *Pparα* which is a target gene of BMAL1 and CLOCK (Oishi et al. 2005) were significantly down-regulated in NPCs from Bmal1^−/−^ mice. Importantly, PPARα agonists upregulate catalase activity (Khoo et al. 2013).

**Fig. 7:**
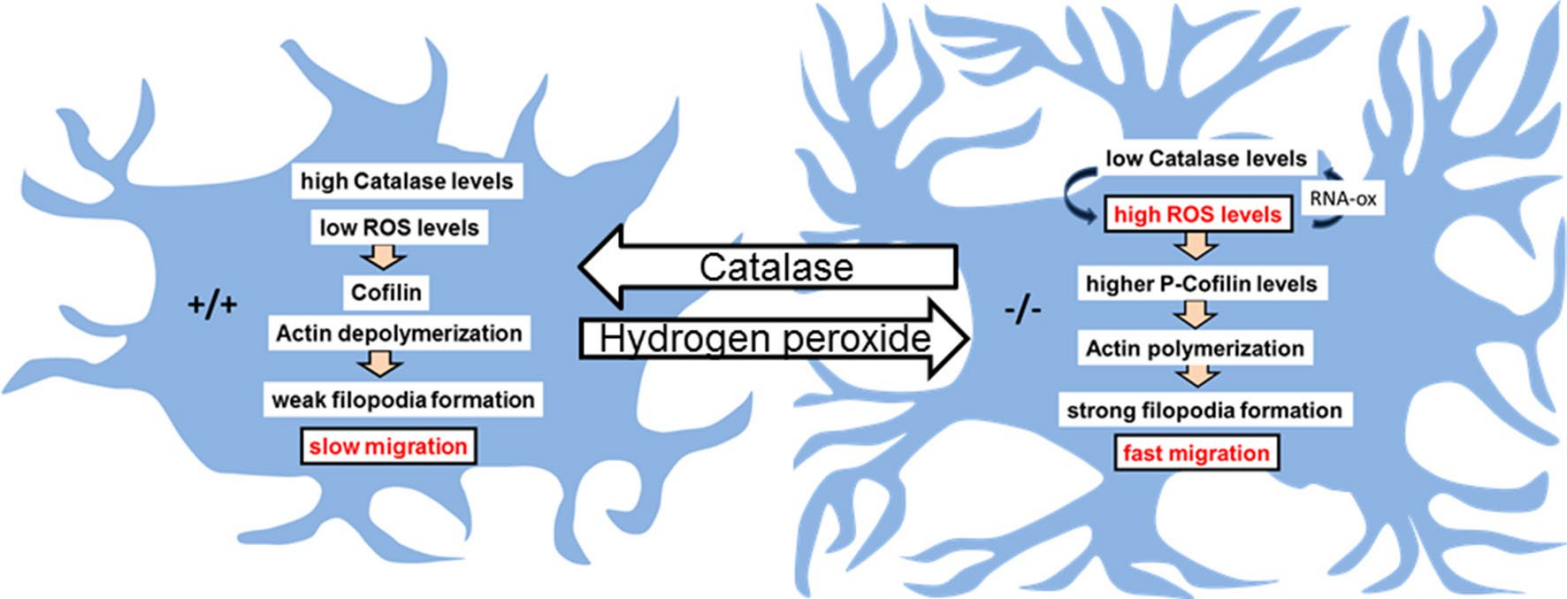
Model for the effect of Bmal1 deficiency on neural progenitor cell (NPC) migration. In NPCs of Bmal1-deficient mice (−/−) impaired detoxification of reactive oxygen species (ROS) results in enhanced RNA oxidation (RNAox). Oxidation of catalase mRNA leads to decreased catalase protein levels and thus further enhancing ROS accumulation. This is associated with a higher level of the ROS-sensitive mediator of actin polymerization p-Cofilin and with stronger filopodia formation and higher migration velocity. Exogenous application of catalase in NPCs from Bmal1^−/−^ mice leads to a reduction of ROS levels and to a wild-type (+/+) filopodia and migration phenotype. Vice versa, exogenous application of hydrogen peroxide in NPCs from Bmal1^+/+^ mice leads to a Bmal1^−/−^ (−/−) filopodia and migration phenotype

Similarly to the in vivo situation, we found an increase in cytoplasmic 8-OH(d)G-Ir in NPCs from Bmal1^−/−^ mice which could be identified as oxidized RNA. Hence, quantification of gene expression in cells compromised by ROS does not necessarily help to predict protein levels as translation of oxidized RNA is affected (Shan et al. 2003). RNA oxidation in NPCs from Bmal1^−/−^ mice was highly specific similarly as shown for ROS-related neuropathological conditions (Shan et al. 2003; Gorg et al. 2008; Nunomura et al. 1999) where it affects signal transmission, neurotransmission, synaptic plasticity, as well as oscillatory networks in the brain (Haussinger and Sies 2013). Thus, our study suggests RNA oxidation as a potential mechanism for cognitive impairment associated with chronic chronodisruption. Moreover, under neuropathological conditions specifically mRNAs encoding for cytoskeleton relevant genes are oxidized (Shan et al. 2003) indicating a potential role of RNA oxidation in modulating cell migration. Oxidation of *Sod2* mRNA was not associated with a decrease in protein levels in Bmal1-deficient mice. However, SOD2 protein has a comparably long half-life (Guruprasad et al. 1990) which is regulated by a deubiquitinating enzyme (Kim et al. 2011). Oxidation of catalase mRNA was associated with decreased respective protein levels consistent with insufficient translation of oxidized mRNA (Shan et al. 2003) and a relatively short half life of the protein (Guruprasad et al. 1990). Decreased catalase activity/level apparently results in accumulation of cellular H_2_O_2_, consistent with a higher susceptibility of Bmal1-deficient primary neurons to H_2_O_2_-induced cell death (Musiek et al. 2013). Treatment of NPCs from Bmal1^−/−^ mice with catalase restored the Bmal1^+/+^ migration phenotype, whereas treatment of NPCs from BMAL1^+/+^ mice with H_2_O_2_ resulted in accelerated migration velocity (Fig. 7). This indicates a relationship between high ROS levels and NPC migration and is consistent with a general cellular “escaping strategy” from unfavorable oxidative environments (Tsirmoula et al. 2015; Hung et al. 2012; Pani et al. 2010). Moreover, H_2_O_2_ is known to affect F-actin polymerization and cell-matrix adhesion (Mocali et al. 1995). *N*-acetylcysteine stimulates glutathione synthesis and migration in fibroblast cell line (Tsai et al. 2014) and has been shown to ameliorate symptoms of premature aging in Bmal1^−/−^ mice (Kondratov et al. 2009). However, treatment of NPCs from Bmal1^−/−^ mice with *N*-acetylcysteine did not affect migration indicating a disulfide bond-independent effect.

In conclusion, disruption of circadian rhythms not only affects brain, (Kondratova et al. 2010) metabolic (Shi et al. 2013) and cardiac (Schroder et al. 2013) function, but also health in general by inducing a pro-inflammatory state (Lucassen et al. 2016). This study provides a novel link between a disturbed molecular clockwork, oxidative stress, RNA oxidation and NPC migration. This is of high significance as it might suggest a general effect of chronodisruption on cell migration in health and disease.

## Electronic supplementary material

Below is the link to the electronic supplementary material.

Figure S1. Representative photomicrographs of cresyl violet-stained parasagittal mouse brain section indicating the analyzed anatomical structures. SVZ, subventricular zone; pRMS, proximal limb of rostral migratory stream, dRMS, distal limb of rostral migratory stream; GCL, granular cell layer; MCL, mitral cell layer, EPL, external plexiform layer, GL, glomerular layer. Scale bar, 200µm (TIF 31213 KB)

Figure S2. Representative photomicrographs of a Bmal1^+/+^ neurospheres. (a) Overview of a neurosphere. The migrating cells were identified as PAS-NCAM-immunoreactive neural progenitors (green) with NucBlue counterstaining (blue) Scale bar= 50 µm (b) perimeter of a neurosphere with migrating PAS-NCAM-immunoreactive neural progenitors (green) at higher magnification. Scale bar= 20 µm. (c) perimeter of a neurosphere with migrating doublecortin (DCX, red)-immunoreactive neural progenitor cells with co-labeling for glial fibrillary acidic protein (GFAP, green) and NucBlue counterstaining (blue) (TIF 27032 KB)

Figure S3. Bmal1 deficiency does not affect the total number of proliferating cells reaching the olfactory bulb within 31 days. Representative photomicrographs and quantification of BrdU^+^ cells (brawn stained cells, black arrows) in the olfactory bulb of Bmal1^+/+^ mice (+/+) and Bmal1^-/-^ mice (-/-). Values are shown as mean +/- SEM. n=5 mice per genotype. Scale bars = 50 µm. Counterstaining with cresyl violet was used to show anatomical location (TIF 26364 KB)

Figure S4. Cytoplasmic 8-OH(d)G immunoreaction represents oxidized RNA. Representative photomicrographs of NPCs from BMAL1^-/-^ mice cytochemically stained with a DNA-marker NucBlue (blue) and immunocytochemically with 8-OH(d)G-antibody (red) 24 h after seeding and treatment with vehicle (control), 10µg/ml DNase I, or 5 µg/µl RNase. Scale bars= 20 µm (TIF 25365 KB)

Figure S5. Treatment of NPCs from Bmal1^-/-^ mice with hydrogen peroxide and NPCs from Bmal1^+/+^ mice with catalase does not affect migration. a) Representative photomicrographs of NPCs from Bmal1^-/-^ mice (-/-) treated with vehicle (control) or 80 µM hydrogen peroxide (H_2_O_2_) for 24 h. Scale bar: 200 µm. (b) Time course of migration distance and velocity is not different between vehicle (control) and treatment with 80 µM H_2_O_2_ during the first 24 h after seeding. n=3 mice per group. (c) Representative photomicrographs of NPCs from Bmal1^++^ mice (+/+) treated with vehicle (control) or 500 U/ml catalase (catalase) for 24 h. Scale bar: 200 µm. (d) Time course of migration distance and velocity is not different between vehicle (control) and treatment with 500 U/ml catalase during the first 24 h after seeding. n=3 mice per group (TIF 30992 KB)

Figure S6 Treatment of NPCs derived from Bmal1^-/-^ mice with *N*-acetylcysteine does not affect migration. Neurospheres derived from Bmal1^-/-^ mice were seeded in migration medium supplemented with different concentrations of *N*-acetylcysteine or vehicle (control) and continuously recorded during the first 24 h (TIF 25289 KB)

Appendix Video 1 Migration of NPCs derived from Bmal1^+/+^ mice. Neurospheres were recorded continuously during the first 24 hours after culture in migration medium. Time interval between phase-contrast images at 100x magnification was 60 min (AVI 38712 KB)

Appendix Video 2 Migration of NPCs derived from Bmal1^-/-^ mice. Neurospheres were recorded continuously during the first 24 hours after seeding in migration medium. Time interval between phase-contrast images at 100x magnification was 60 min (AVI 38574 KB)

Appendix Video 3 Migration of NPCs derived from Bmal1^+/+^ mice in the presence of hydrogen peroxide. Neurospheres were recorded continuously during the first 24 hours after seeding in migration medium in the presence of 80 µM hydrogen peroxide (H_2_O_2_). Time interval between phase contrast images at 100x magnification was 60 min (AVI 38634 KB)

Appendix Video 4 Migration of NPCs derived from Bmal1^-/-^ mice in the presence of catalase. Neurospheres were recorded continuously during the first 24 hours after seeding in migration medium in the presence of 500 U/ml catalase. Time interval between phase contrast images at 100x magnification was 60 min (AVI 38072 KB)
