# Supplementary figures and images for: Deficiency of the clock gene Bmal1 affects neural progenitor cell migration
Source: Brain Struct Funct. 2018 Oct 19;224(1):373–86. doi: 10.1007/s00429-018-1775-1 (PMC6373387; doi:10.1007/s00429-018-1775-1)

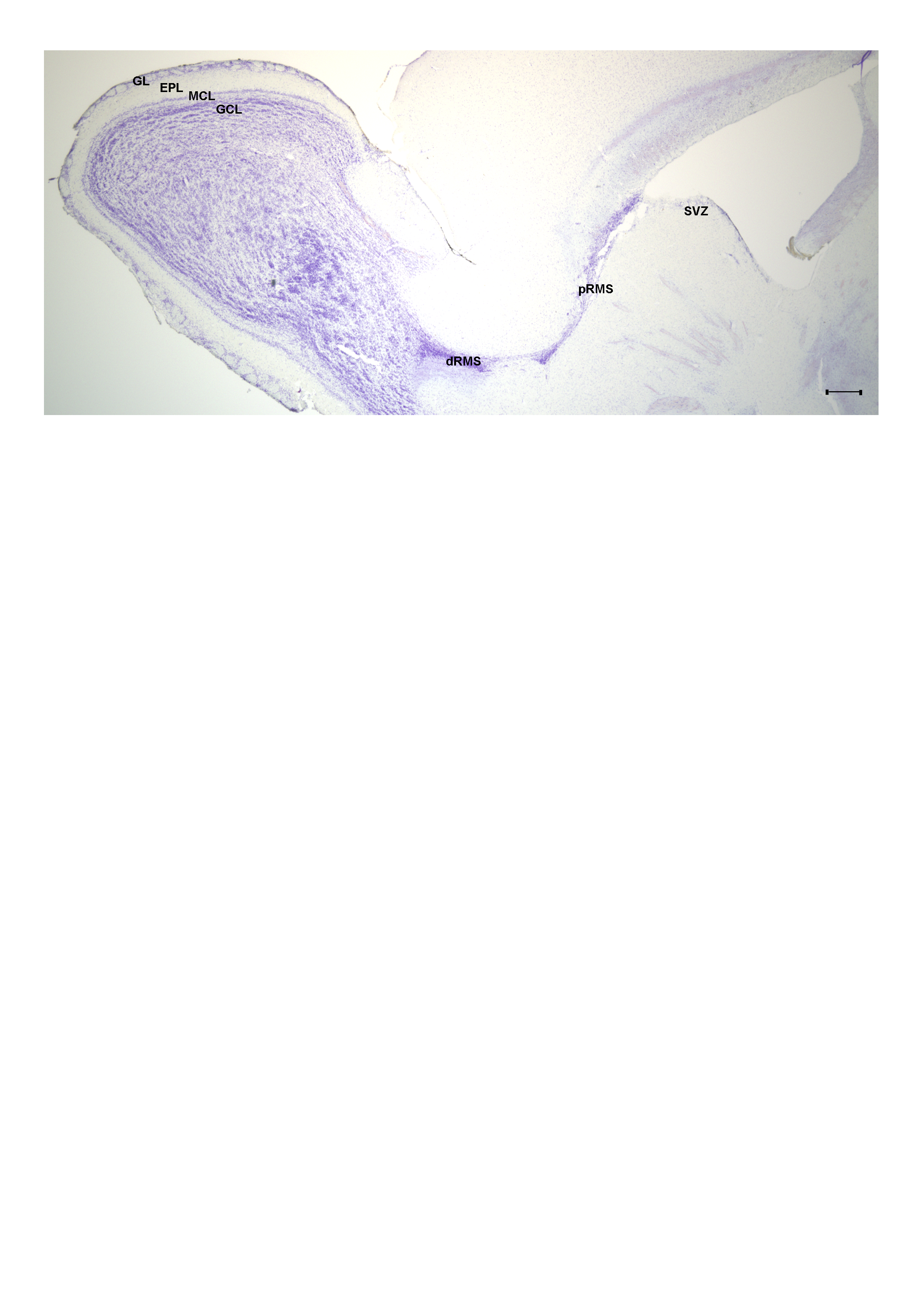

Supplement: Supplementary file 1 — Figure S1. Representative photomicrographs of cresyl violet-stained parasagittal mouse brain section indicating the analyzed anatomical structures. SVZ, subventricular zone; pRMS, proximal limb of rostral migratory stream, dRMS, distal limb of rostral migratory stream; GCL, granular cell layer; MCL, mitral cell layer, EPL, external plexiform layer, GL, glomerular layer. Scale bar, 200µm (TIF 31213 KB) [file 429_2018_1775_MOESM1_ESM.tif]

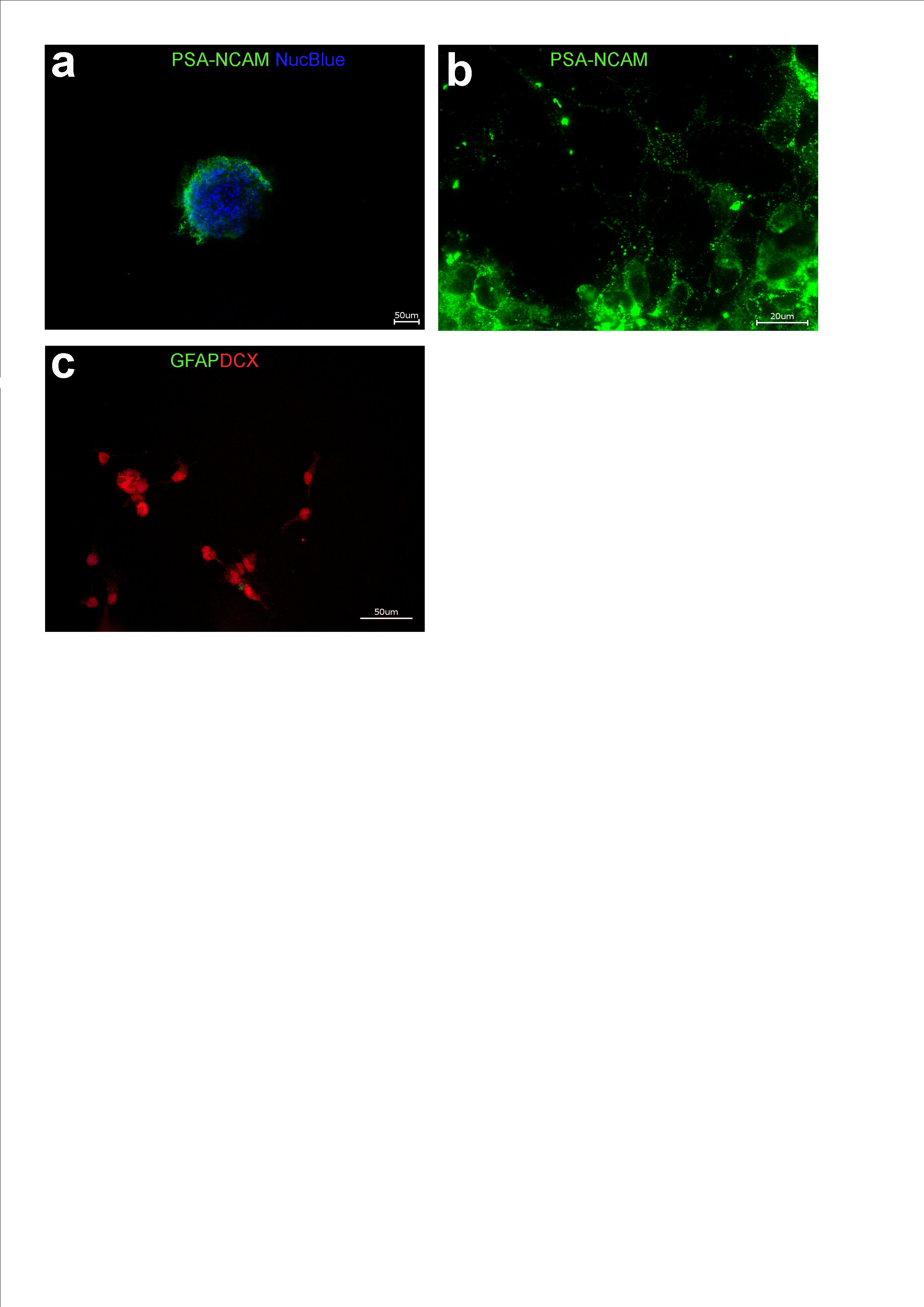

Supplement: Supplementary file 2 — Figure S2. Representative photomicrographs of a Bmal1+/+ neurospheres. (a) Overview of a neurosphere. The migrating cells were identified as PAS-NCAM-immunoreactive neural progenitors (green) with NucBlue counterstaining (blue) Scale bar= 50 µm (b) perimeter of a neurosphere with migrating PAS-NCAM-immunoreactive neural progenitors (green) at higher magnification. Scale bar= 20 µm. (c) perimeter of a neurosphere with migrating doublecortin (DCX, red)-immunoreactive neural progenitor cells with co-labeling for glial fibrillary acidic protein (GFAP, green) and NucBlue counterstaining (blue) (TIF 27032 KB) [file 429_2018_1775_MOESM2_ESM.tif]

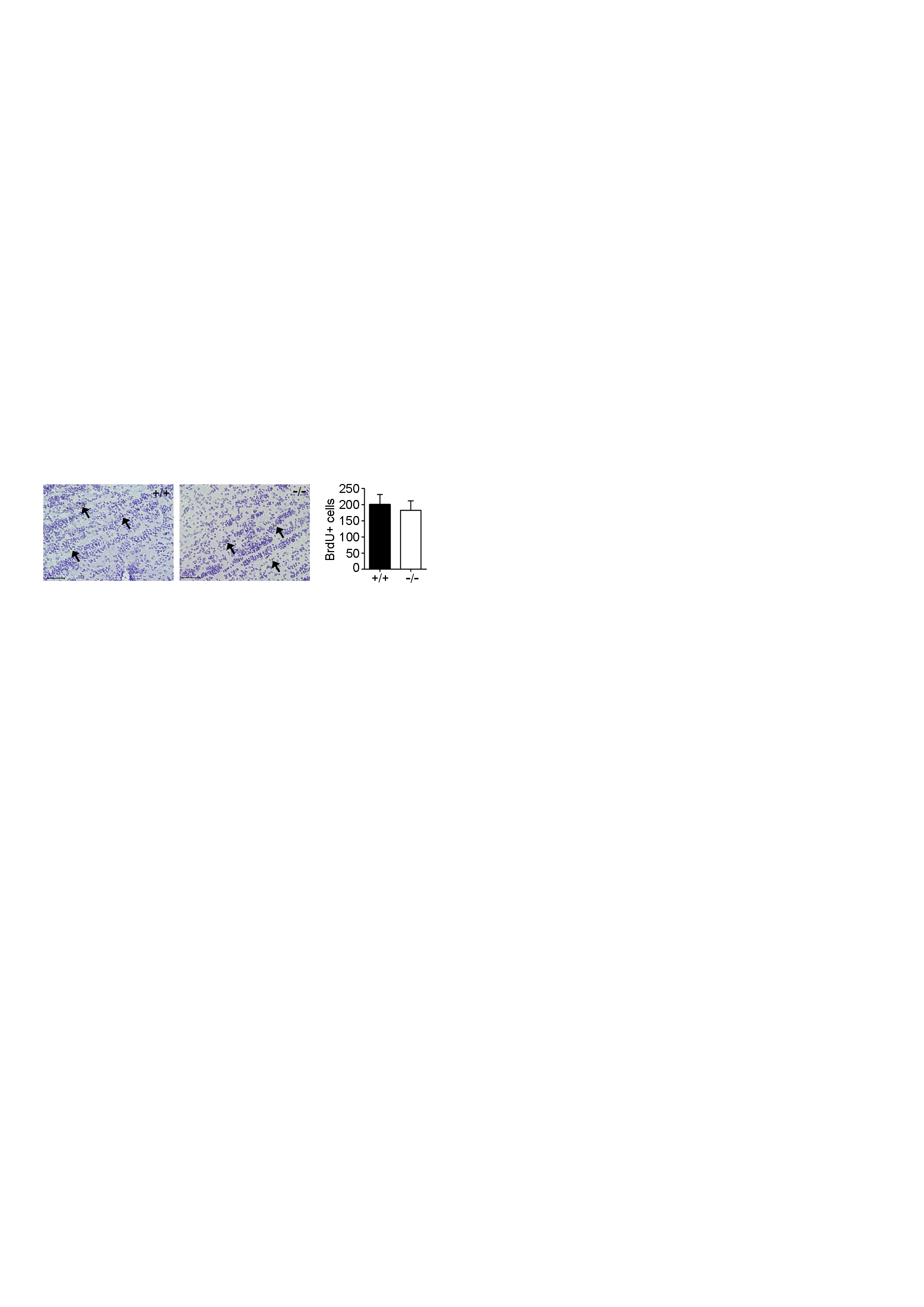

Supplement: Supplementary file 3 — Figure S3. Bmal1 deficiency does not affect the total number of proliferating cells reaching the olfactory bulb within 31 days. Representative photomicrographs and quantification of BrdU+ cells (brawn stained cells, black arrows) in the olfactory bulb of Bmal1+/+ mice (+/+) and Bmal1-/- mice (-/-). Values are shown as mean +/- SEM. n=5 mice per genotype. Scale bars = 50 µm. Counterstaining with cresyl violet was used to show anatomical location (TIF 26364 KB) [file 429_2018_1775_MOESM3_ESM.tif]

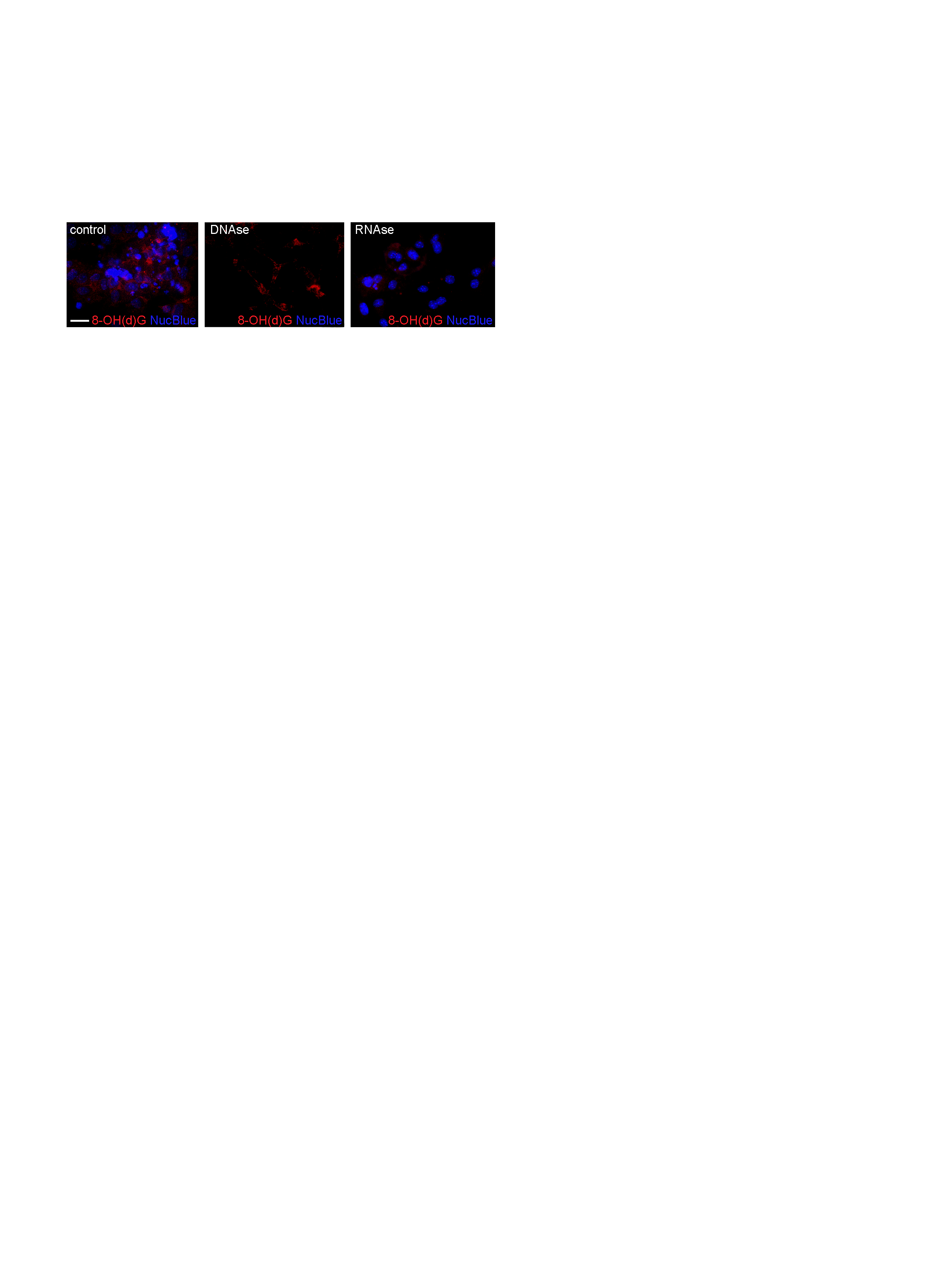

Supplement: Supplementary file 4 — Figure S4. Cytoplasmic 8-OH(d)G immunoreaction represents oxidized RNA. Representative photomicrographs of NPCs from BMAL1-/- mice cytochemically stained with a DNA-marker NucBlue (blue) and immunocytochemically with 8-OH(d)G-antibody (red) 24 h after seeding and treatment with vehicle (control), 10µg/ml DNase I, or 5 µg/µl RNase. Scale bars= 20 µm (TIF 25365 KB) [file 429_2018_1775_MOESM4_ESM.tif]

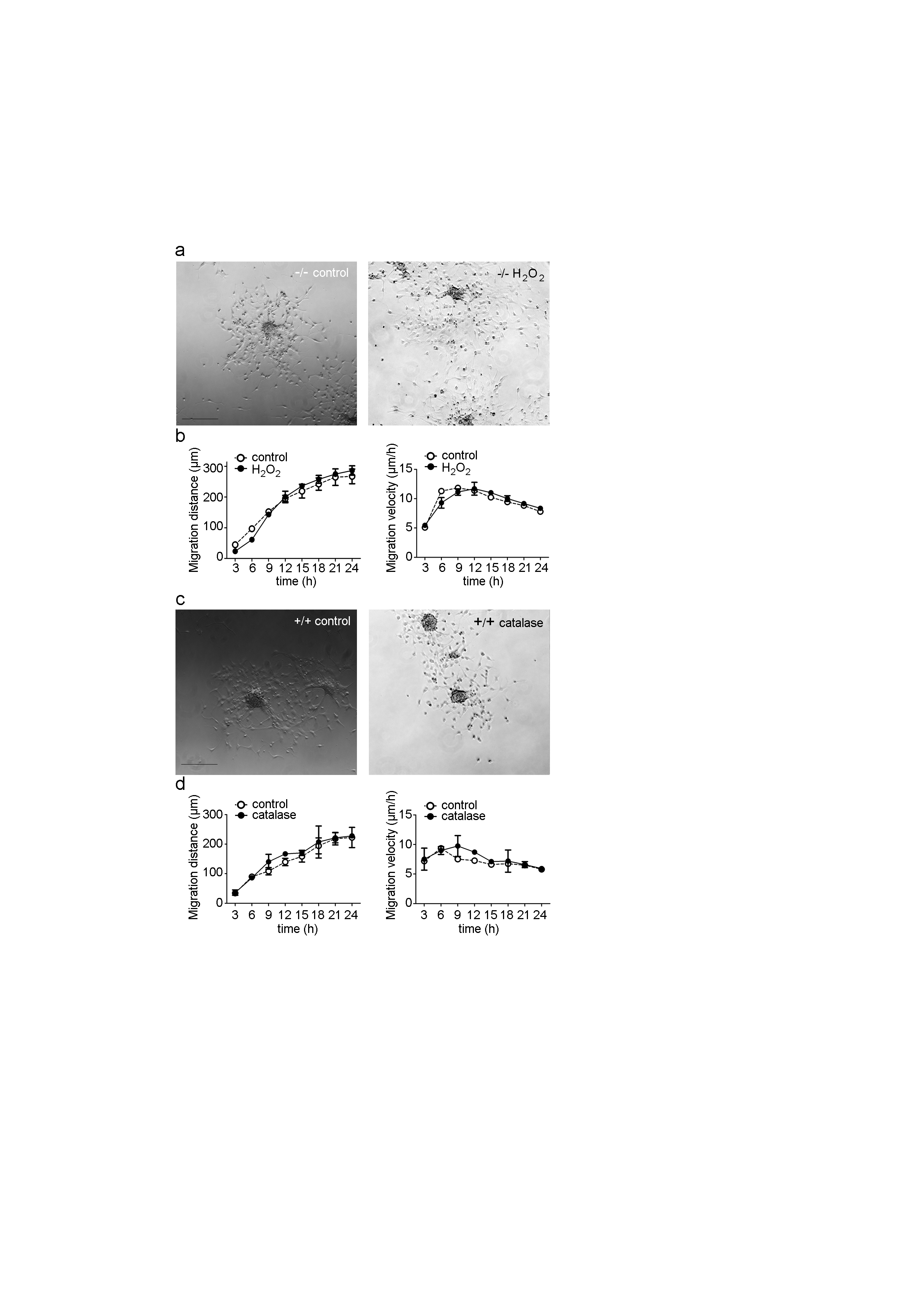

Supplement: Supplementary file 5 — Figure S5. Treatment of NPCs from Bmal1-/- mice with hydrogen peroxide and NPCs from Bmal1+/+ mice with catalase does not affect migration. a) Representative photomicrographs of NPCs from Bmal1-/- mice (-/-) treated with vehicle (control) or 80 µM hydrogen peroxide (H2O2) for 24 h. Scale bar: 200 µm. (b) Time course of migration distance and velocity is not different between vehicle (control) and treatment with 80 µM H2O2 during the first 24 h after seeding. n=3 mice per group. (c) Representative photomicrographs of NPCs from Bmal1++ mice (+/+) treated with vehicle (control) or 500 U/ml catalase (catalase) for 24 h. Scale bar: 200 µm. (d) Time course of migration distance and velocity is not different between vehicle (control) and treatment with 500 U/ml catalase during the first 24 h after seeding. n=3 mice per group (TIF 30992 KB) [file 429_2018_1775_MOESM5_ESM.tif]

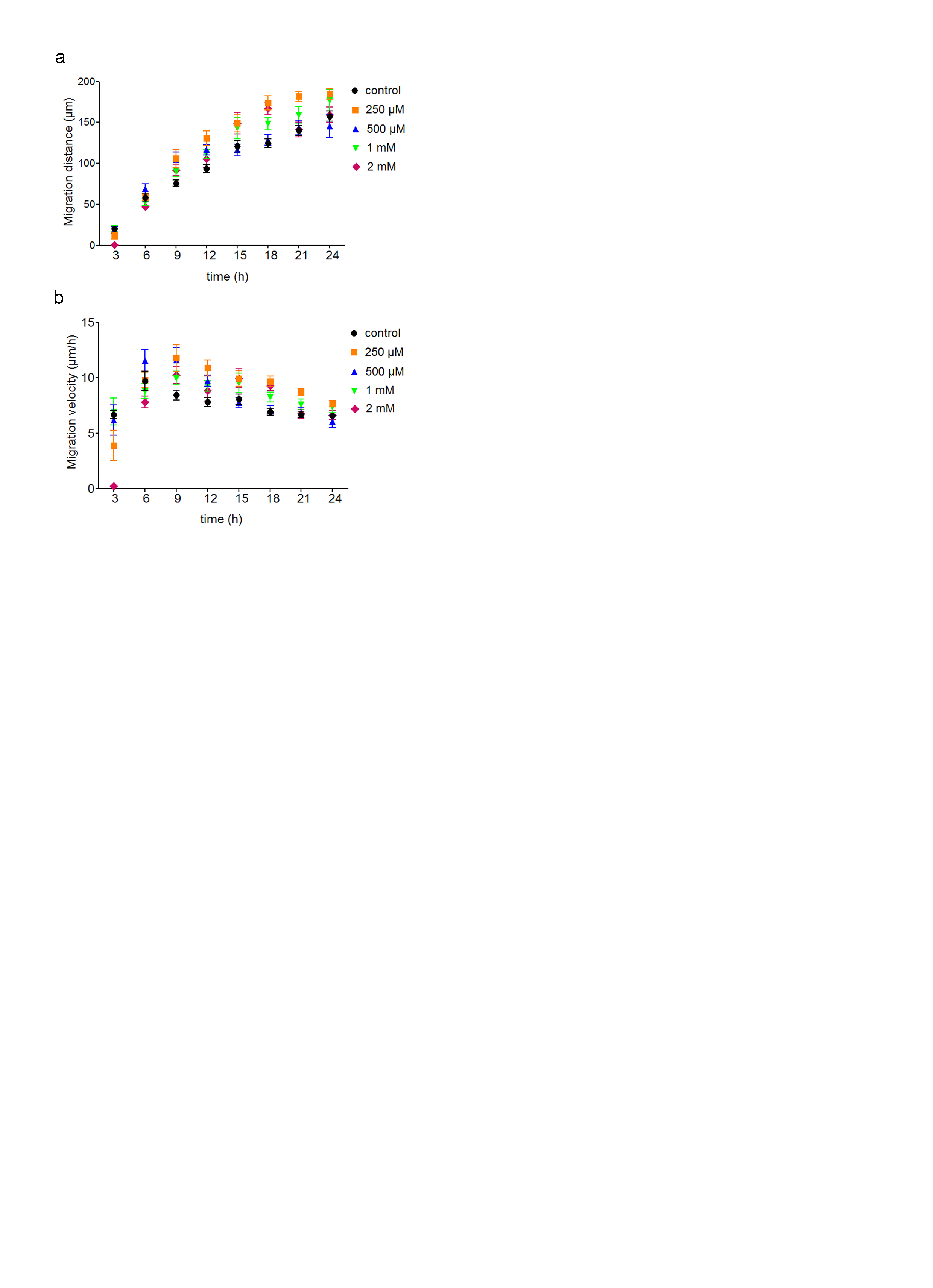

Supplement: Supplementary file 6 — Figure S6 Treatment of NPCs derived from Bmal1-/- mice with N-acetylcysteine does not affect migration. Neurospheres derived from Bmal1-/- mice were seeded in migration medium supplemented with different concentrations of N-acetylcysteine or vehicle (control) and continuously recorded during the first 24 h (TIF 25289 KB) [file 429_2018_1775_MOESM6_ESM.tif]
